# Supplementary material for: Non-Thermal Plasma Induces Antileukemic Effect Through mTOR Ubiquitination
Source: Cells. 2020 Mar 2;9(3):595. doi: 10.3390/cells9030595 (PMC7140413; doi:10.3390/cells9030595)
Supplement: Supplementary file 1 [file cells-09-00595-s001.pdf]

Supplementary Information

Non-Thermal Plasma induces antileukemic effect through mTOR ubiquitination

Sun-Yong Kim[1,2*], Hyo Jeong Kim[1,2,3*], Haeng Jun Kim[1,2,3], and Chul-Ho Kim[1,2,3]

[1]Department of Otolaryngology, Ajou University School of Medicine, Suwon, Republic of Korea

[2]Oncoprotein Modification and Regulation Research Center

[3]Department of Molecular Science and Technology, Ajou University, Suwon, Republic of Korea

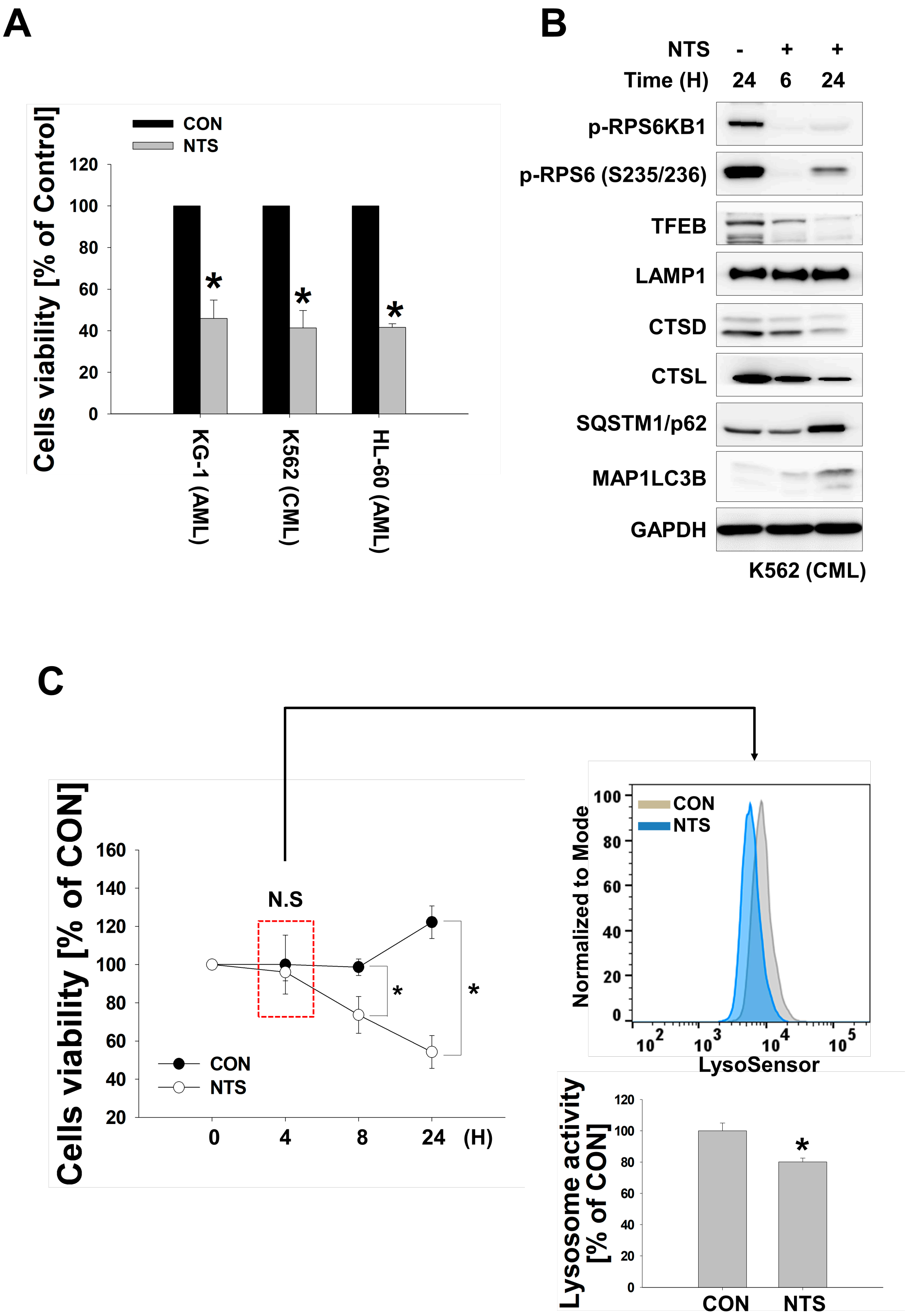

Figure S1. NTS induces leukemia cell death. (**A**) AML cells (KG-1 and HL-60) or CML cells (K562) were treated with NTS containing 10% FBS for 24 hours. NTS-induced

cytotoxicity was determined by MTS assay. Data are means ± SD. Asterisks indicate statistically significant differences (n = 6, *P < 0.05). (**B**) Lysosomal proteins are downregulated by NTS. K562 cells were treated with NTS for the indicated times (6 and 24 hours) and protein levels were analyzed by western Blot. (**C**) Lysosome inhibition is preceded under NTS-induced cell death. K562 cells were treated with NTS for the indicated times (4, 8, and 24 hours) and cell viability was observed by MTS assay. Data are means ± SD. Asterisks indicate statistically significant differences (n = 6, *P < 0.05). After the treatment with NTS for 4 hours, cells were labeled with LysoSensor green. The total fluorescence intensity of LysoSensor was measured using FACS analysis. Data are means ± SD. Asterisks indicate statistically significant differences (n = 3, *P < 0.05).

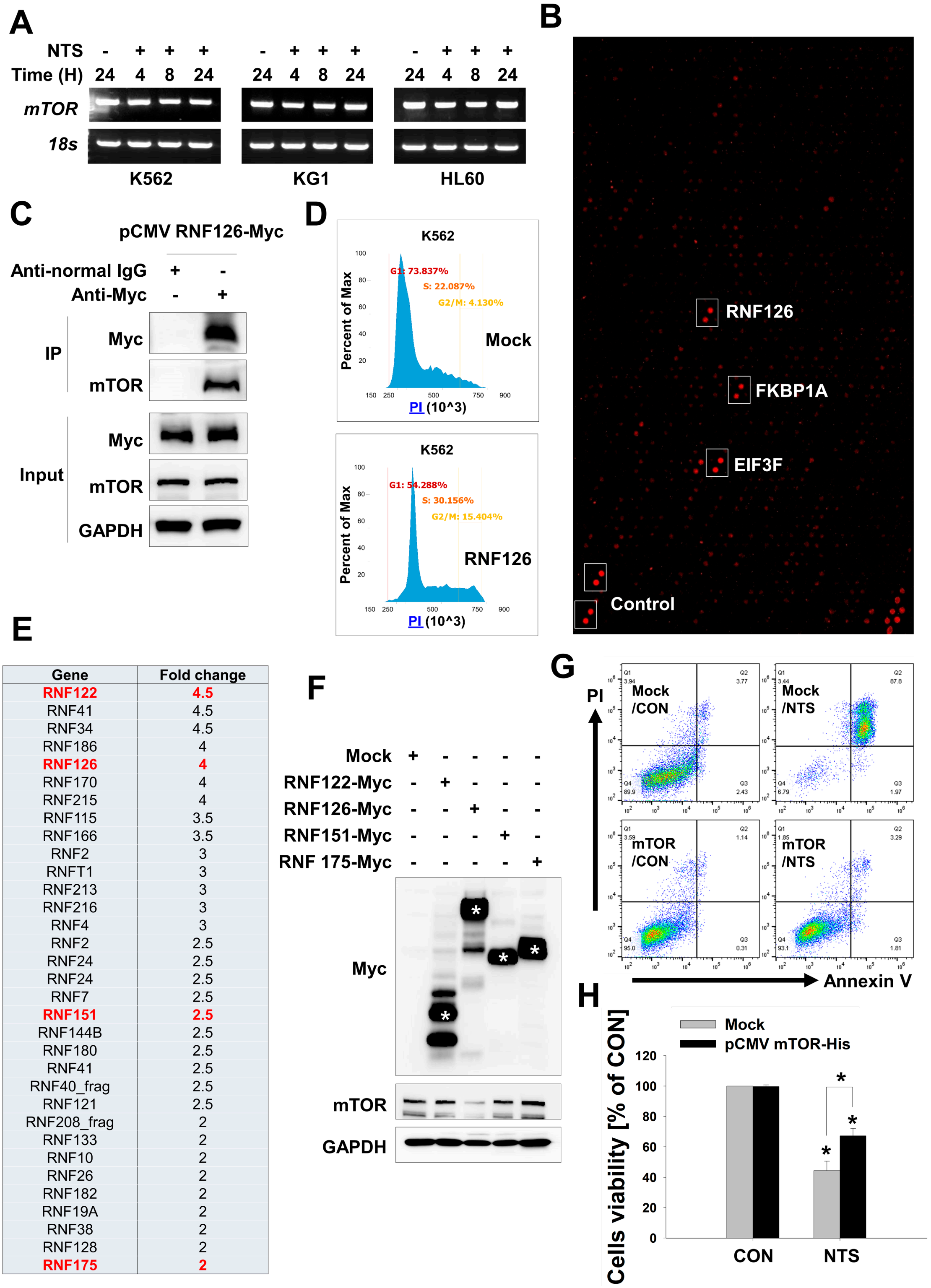

Figure S2. RNF126 acts as negative regulator of mTOR. (A) *mTOR* gene expression is not changed by NTS treatment. Leukemia cells were treated with NTS for the indicated times (4, 8, and 24 hours). NTS treatment was given for each indicated time to leukemia

cells and then *mTOR* gene expression level was determined by RT-PCR. (**B**) Identification of novel binding proteins for mTOR. RNF126 was selected among novel interacting protein for mTOR by protein microarray. FKBP1A or EIF3F was used as positive interacting proteins for mTOR. (**C**) RNF126 interacts with mTOR. K562 cells were transiently transfected with a plasmid expressing pCMV RNF126-Myc. After 24 hours, cell lysates were immunoprecipitated with anti-normal IgG or anti-Myc antibody. (**D**) RNF126 overexpression induces G2/M cell cycle arrest in K562 cells. K562 cells were transfected with Mock or pCMV RNF126-Myc for 48 hours and cell cycle analysis was processed after 70% ethanol fixation and PI staining. Cell cycle was measured by FACS analysis. (**E**) The list of RNF family proteins. K562 cells were treated for overnight and then, gene expression level change was analyzed by DNA microarray. Over 2-fold upregulated RNF family proteins were listed. (**F**) mTOR is regulated by only RNF126. K562 cells were transfected with NTS-induced up-regulated RNF family plasmids and then, each indicated proteins level was evaluated by western blot (* means overexpressed proteins of each RNF family proteins). (**G** and **H**) mTOR overexpression prevents NTS-induced cell death. K562 cells were transfected with mTOR-His plasmid. After 24 hours, cells were treated with NTS for 24 hours, and then cells were stained with Annexin V/PI (**G**) or cells viability was measured by MTS assay (**H**). Data are means ± SD. Asterisks indicate statistically significant differences (n=6, *$P < 0.05$).

A

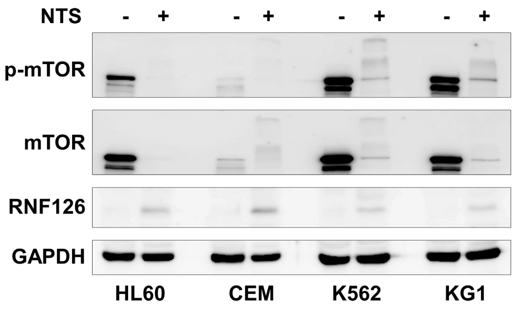

B

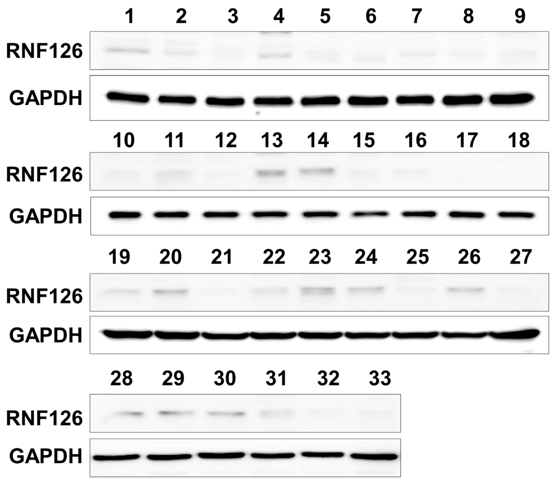

Number	Cell-line	Origen
1	Cal27	head & neck cancer (HNC)
2	SCC15	HNC
3	SCC25	HNC
4	SNU1483	HNC
5	SCCQLL1	HNC
6	MSKQLL1	HNC
7	FaDu	HNC
8	SNU1041	HNC
9	8505C	thyroid
10	C643	thyroid
11	OE33	esophageal
12	PC12	adrenal gland
13	U251	glioma
14	U87MG	glioma
15	SNU16	gastric
16	SNU638	gastric
17	AGS	gastric
18	MKN28	gastric
19	HepG2	liver
20	Huh7	liver
21	SNU475	liver
22	H1975	lung
23	A549	lung
24	H1993	lung
25	HCT116	colon
26	HT29	colon
27	HT1080	sarcoma
28	RD	sarcoma
29	HeLa	cervix
30	MDA-MB-231	breast
31	MKN45	gastric
32	U266	multiple myeloma
33	K562	leukemia

Figure S3. RNF126 expression is suppressed in cancer cell lines. (**A**) RNF126 is upregulated under NTS treatment. Cells were cultured with or without NTS for 24 hours, after protein levels were determined by western Blot. (**B**) Endogenous RNF126 expression profiling was evaluated by western Blot (*left*) and cell-lines list was showed in table (*right*).

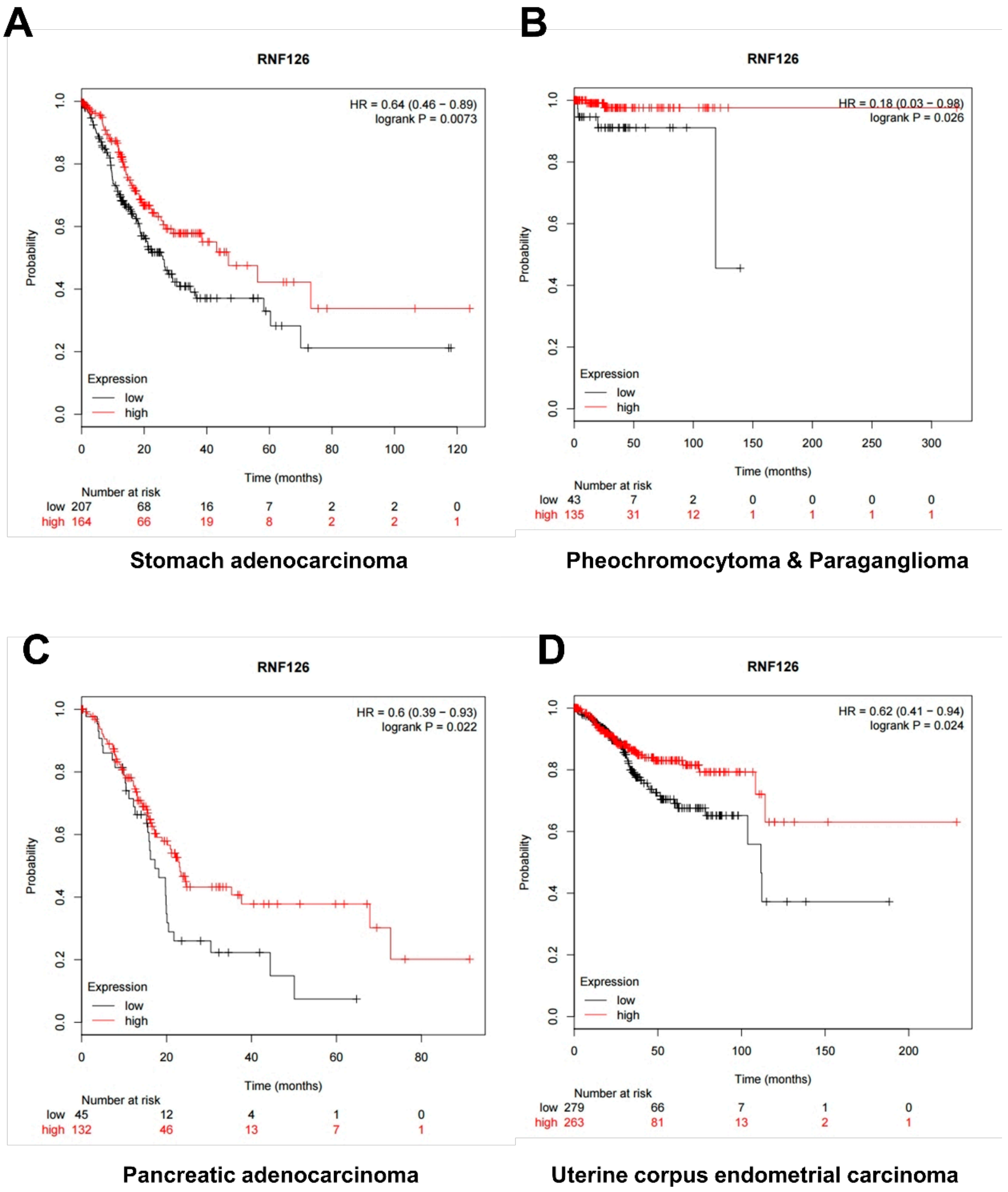

Figure S4. Low expression of RNF126 shows poor survival ratio in TCGA data. Hight expression level of RNF126 was showed good survival ratio rather than low expression group in Stomach adenocarcinoma (**A**), Pheochromocytoama & Paraganglioma (**B**), Pancreatic adenocarcinoma (**C**) or uterine corpus endometrial carcinoma (**D**).

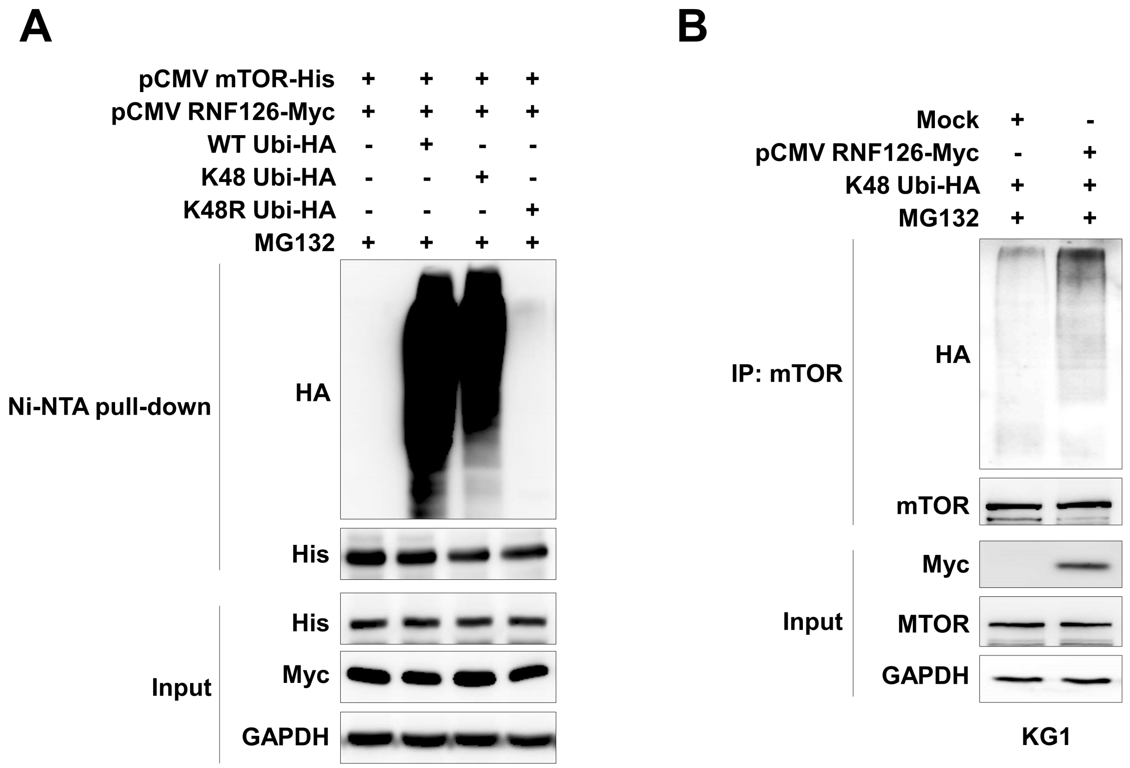

Figure S5. RNF126 induces K48-linked ubiquitination of mTOR. (**A**) K562 cells were co-transfected with pCMV mTOR-His, pCMV RNF126-Myc, WT Ubi-HA, K48 Ubi-HA, or K48R Ubi-HA for 24 hours. Cells were treated with MG132 (10 μM) for 6 hours before harvest, and then subjected to Ni-NTA His pull-down for ubiquitination assay. (**B**) KG1 cells were co-transfected with pCMV RNF126-Myc and K48 Ubi-HA for 24 hours. Cells were treated with MG132 (10 μM) for 6 hours before harvest, and then subjected to immunoprecipitation with anti-mTOR antibody.

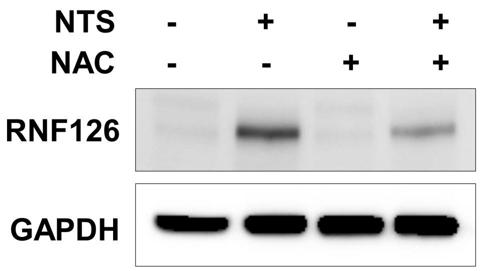

Figure S6. NAC inhibits NTS-induced RNF126 expression. K562 cells were pre-treated with NAC for 1 hour and NTS was treated further 24 hours. RNF126 level was determined by western Blot.

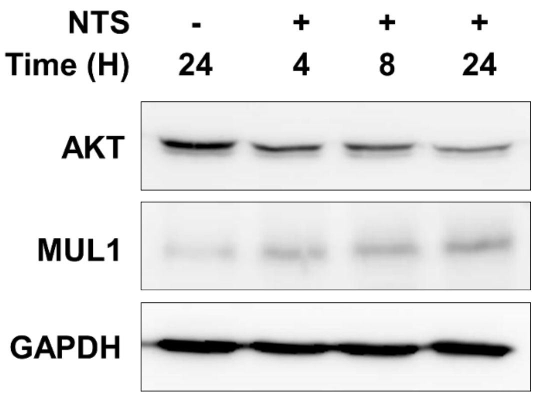

Figure S7. AKT/MUL1 axis is associated in NTS-induced antileukemic effects. K562 cells were treated with NTS for the indicated times (4, 8, and 24 hours). AKT or MUL1 expression levels were analyzed by western Blot.
